# New insight into the function of wheat glutenin proteins as investigated with two series of genetic mutants

**DOI:** 10.1038/s41598-017-03393-6

**Published:** 2017-06-13

**Authors:** Zhaojun Wang, Yiwen Li, Yushuang Yang, Xin Liu, Huanju Qin, Zhenying Dong, Shuhai Zheng, Kunpu Zhang, Daowen Wang

**Affiliations:** 1 The State Key Laboratory of Plant Cell and Chromosome Engineering, Institute of Genetics and Developmental Biology, Chinese Academy of Sciences, Beijing, 100101 China; 2 University of Chinese Academy of Sciences, Beijing, 100049 China; 3 Zhaoxian Institute of Agricultural Sciences, Zhaoxian, 051530 China; 4 The Collaborative Innovation Center for Grain Crops, Henan Agricultural University, Zhengzhou, 450002 China

## Abstract

Among the three major food crops (rice, wheat and maize), wheat is unique in accumulating gluten proteins in its grains. Of these proteins, the high and low molecular weight glutenin subunits (HMW-GSs and LMW-GSs) form glutenin macropolymers that are vital for the diverse end-uses of wheat grains. In this work, we developed a new series of deletion mutants lacking one or two of the three *Glu-1* loci (*Glu-A1*, *-B1* and -*D1*) specifying HMW-GSs. Comparative analysis of single and double deletion mutants reinforced the suggestion that *Glu-D1* (encoding the HMW-GSs 1Dx2 and 1Dy12) has the largest effects on the parameters related to gluten and dough functionalities and breadmaking quality. Consistent with this suggestion, the deletion mutants lacking *Glu-D1* or its combination with *Glu-A1* or *Glu-B1* generally exhibited strong decreases in functional glutenin macropolymers (FGMPs) and in the incorporation of HMW-GSs and LMW-GSs into FGMPs. Further examination of two knockout mutants missing 1Dx2 or 1Dy12 showed that 1Dx2 was clearly more effective than 1Dy12 in promoting FGMPs by enabling the incorporation of more HMW-GSs and LMW-GSs into FGMPs. The new insight obtained and the mutants developed by us may aid further research on the control of wheat end-use quality by glutenin proteins.

## Introduction

Wheat is one of the major food crops in the world. Compared with other major cereals like rice and maize, wheat has unique end-use traits that are important for making a variety of globally consumed foods, such as various types of bread and noodles^1^. Two main families of gluten proteins, i.e., glutenins and gliadins, are involved in the formation of wheat end-use quality. The glutenins can be further divided into two subfamilies, high molecular weight glutenin subunits (HMW-GSs) and low molecular weight glutenin subunits (LMW-GSs), while gliadins contain four subfamilies, α/β-, γ-, δ- and ω-gliadins^2–4^. During dough processing, these proteins form a complex network (i.e., gluten), which confers viscoelasticity to the dough. The viscoelastic property of a dough determines its suitability for making a particular type of wheat food^2, 5^. Thus, variations in the relative amount and composition of glutenins and gliadins have important effects on gluten functionality, dough viscoelasticity, and end-use quality^6, 7^.

In common wheat (*Triticum aestivum*, 2n = 6x = 42), HMW-GSs are encoded by three homoeologous loci (*Glu-A1*, *Glu-B1* and *Glu-D1*) on the long arms of group 1 chromosomes^8^. In each locus, there exist two HMW-GS genes, encoding a x- and a y-type subunits, respectively^7, 9^. Because of gene silencing and allelic variation, three to five HMW-GSs are usually expressed in common wheat, with HMW-GS composition often differing among different cultivars^2, 8^. The LMW-GSs are encoded by *Glu-A3*, *Glu-B3* and *Glu-D3* loci on the short arms of group 1 chromosomes^10^. In general, each *Glu-3* locus contain several LMW-GS genes highly similar in nucleotide sequence and expression pattern, and each LMW-GS gene member frequently has two or more alleles^11, 12^. Gliadin genes are located in six major chromosomal loci, with *Gli-A1*, *-B1*, *-D1* on the short arms of group 1 chromosomes and *Gli-A2*, *-B2* and *-D2* on the short arms of group 6 chromosomes^13, 14^. Some minor gliadin loci, e.g., *Gli-3* and *Gli-5*, have also been reported on the short arms of group 1 chromosomes^15, 16^. Although the precise number of genes specifying gliadins is still unknown at present, it is generally accepted that the genes expressing gliadins are substantially more than those producing glutenins. Consequently, in bread wheat, HMW-GSs, LMW-GSs and total gliadins account for 7–15%, 20–35% and 40–50% of the gluten proteins, respectively^1, 6, 17^.

In the gluten complex, HMW-GSs and LMW-GSs covalently interact with each other by inter-molecular disulfide bonds, thus exist as glutenin macropolymers (GMPs)^18–21^. Gliadins exist mainly as monomers, and interact non-covalently with GMPs^7, 17, 22^. However, the gliadin carrying an odd number of cysteine residues can also take part in the formation of GMPs via inter-molecular disulfide bond^23^. Previous studies have shown that GMPs, especially those with a molecular mass greater than 250 kD, are the key determinant of gluten and dough functionalities and end-use quality^6, 17, 24, 25^. Gliadins may act as plasticizer to modify the extensibility of gluten and dough and thus the end-use traits^14, 26, 27^. The GMPs with larger molecular mass, i.e., functional GMPs (FGMPs), are insoluble in the protein extraction buffers without reductant, and can be separated from the smaller polymers and monomers and measured quantitatively. The sodium dodecyl sulfate (SDS) unextractable polymeric protein (UPP) fraction^28–30^ and the 50% (v/v) 1-propanol insoluble glutenin (IG) preparation^31–33^ are reliable indicators of FGMPs because both have been proved to be significantly and strongly related to wheat end-use quality. The protocol for IG preparation also allows the fractionation of soluble glutenin (SG) polymers that have comparatively smaller molecular mass^31^. Since both IG and SG consist of HMW-GSs and LMW-GSs^31^, the relative amounts of the two fractions reflect the behavior of glutenin subunits during their polymerization into GMPs.

Many studies aimed to dissect the structure of GMP have been carried out. Regarding HMW-GSs, only x-x and x-y dimers were found, but not those consisting of solely y-type HMW-GSs^34–37^. LMW-GSs could aggregate solely to form smaller polymers, and these polymers interacted covalently with y-type HMW-GSs by disulfide bonds^18, 38^. Mapping the disulfide bonds formed between glutenin subunits using mass spectrometry confirmed the existence of x-x and x-y HMW-GS interactions^23, 39–42^, and verified the disulfide bond formed by LMW-GSs and y-type HMW-GSs^41^. Therefore, in the current model on the main structure of GMP, HMW-GSs act as the backbone, with LMW-GSs as branches through bonding to y-type HMW-GSs^43^. This structural model is consistent with the dominant effects of HMW-GSs over LMW-GSs on wheat end-use quality. It has been widely observed that HMW-GSs, though occupying only 7–15% of the total gluten proteins, can explain 45–70% of the variations of breadmaking performance^5, 44, 45^.

Despite the studies described above, there remain important gaps in the understanding of the action of HMW-GSs and LMW-GSs in controlling wheat end-use quality. Although it is known that the importance of three *Glu-1* loci in end-use quality control is *Glu-D1* > *Glu-B1* > *Glu-A1* ^46–49^, the mechanism causing such difference is still not very clear. Since *Glu-D1* specifies one x- and one y-type HMW-GSs, it is logic to ask if the two subunits may differ in their effects on wheat end-use quality. And if so, what is the underlying mechanism? Answering these questions will deepen our understanding of the genetic and molecular basis of wheat end-use quality, thus increasing the effectiveness in improving related processing traits. Therefore, the main objective of this study was to gather new information on the function of HMW-GSs and LMW-GSs in controlling important gluten, dough and breadmaking quality parameters using two sets of genetic mutants. The first set lacked one or two of the three *Glu-1* loci, with the double mutants being developed using the single mutants that we published in a previous study^49^. The second set included two ethyl methanesulfonate (EMS) induced knockout mutants lacking *Glu-D1* specified HMW-GSs 1Dx2 and 1Dy12, respectively^45^. Considering that the gluten, dough and end-use quality traits of common wheat are generally controlled by polygenes, and affected by the environments^22, 28, 50, 51^, the mutants and their wild type (WT) progenitors were cultivated in multiple environments. The use of the resultant grain samples facilitated a more objective assessment of the effects of different genetic mutations on representative gluten, dough and breadmaking parameters. The observed effects were then related to the changes in GMP, UPP, IG and SG in order to explore the mechanisms involved. Finally, the new insight gained and its implications for understanding the gluten protein interactions in wheat end-use quality control are discussed.

## Results

### Development of double mutant deletion lines

**Figure 1:**
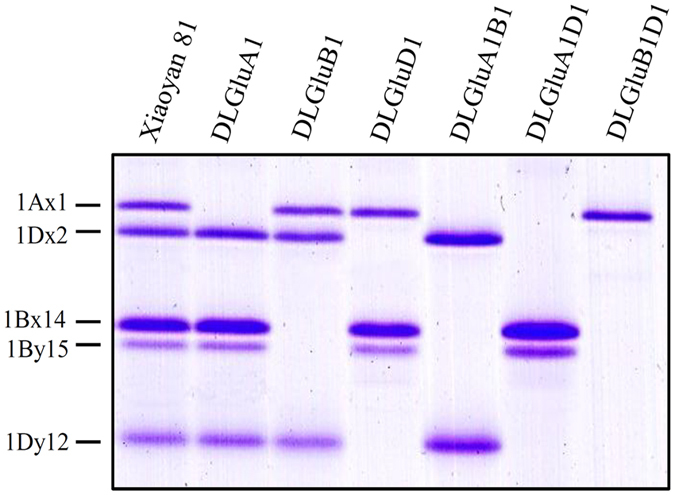
Composition of HMW-GSs in Xiaoyan 81 and six derivative deletion mutants. Xiaoyan 81 is the wild type progenitor, has all three *Glu-1* loci (*Glu-A1*, *-B1* and *-D1*), and expresses five HMW-GSs (1Ax1, 1Dx2, 1Bx14, 1By15 and 1Dy12). DLGluA1, DLGluB1 and DLGluD1 are single deletion mutants lacking *Glu-A1*, *-B1* and *-D1*, respectively. DLGluA1B1, DLGluA1D1 and DLGluB1D1 are double deletion mutants lacking *Glu-A1*/*Glu-B1*, *Glu-A1*/*Glu-D1* and *Glu-B1*/*Glu-D1*, respectively. The HMW-GSs missed in the six mutants are 1Ax1 (DLGluA1), 1Bx14 + 1By15 (DLGluB1), 1Dx2 + 1Dy12 (DLGluD1), 1Ax1 + 1Bx14 + 1By15 (DLGluA1B1), 1Ax1 + 1Dx2 + 1Dy12 (DLGluA1D1), and 1Bx14 + 1By15 + 1Dx2 + 1Dy12 (DLGluB1D1), respectively.

In our previous work, three ion beam induced deletion lines (DLGluA1, DLGluB1 and DLGluD1) missing *Glu-A1*, *-B1* and *-D1*, respectively, were created^49^. Here, by conducting appropriate crossing among DLGluA1, DLGluB1 and DLGluD1, three double mutant deletion lines, DLGluA1B1 (lacking *Glu-A1* and *-B1*), DLGluA1D1 (missing *Glu-A1* and *-D1*) and DLGluB1D1 (devoid of *Glu-B1* and *-D1*) were developed. SDS-PAGE analysis of seed proteins confirmed that DLGluA1B1, DLGluA1D1 and DLGluB1D1 lacked three (1Ax1, 1Bx14 and 1By15), three (1Ax1, 1Dx2 and 1Dy12) and four (1Bx14, 1By15, 1Dx2 and 1Dy12) HMW-GSs, respectively, whereas the five subunits were all normally accumulated in the wild type (WT) progenitor Xiaoyan 81 (Fig. 1). In general, the six deletion lines did not differ substantially from the WT control (Xiaoyan 81) in plant height, tiller number, grain number per spike, grain length, grain weight, and thousand kernel weight under field conditions (Table S1).

### Changes of gluten, dough and end-use quality parameters

Xiaoyan 81 and the six deletion lines were cultivated in five field environments in two wheat crop seasons (2013/2014 and 2014/2015). The grains were harvested and milled, and the resultant flour samples were examined for representative gluten, dough and end-use quality parameters. In the two environments (ZX and XX) in 2013/2014, Zeleny sedimentation value (ZSV) and three Mixograph parameters, i.e., midline peak time (MPT), midline peak height (MPH) and midline peak width (MPW), were measured. ZSV is a reliable and commonly used indicator of gluten strength, with a higher ZSV indicating stronger gluten^52, 53^. MPT, MPH and MPW reflect dough property, with stronger and more elastic dough having higher values in the three parameters^54, 55^. Relative to Xiaoyan 81, the four parameters were all significantly decreased in the six deletion lines in both environments (Table 1). Generally, the decrease was strongest in DLGluD1, DLGluA1D1 and DLGluB1D1, intermediate in DLGluA1B1, and relatively low in DLGluA1 and DLGluB1 (Table 1).

**Table 1:** Comparison of ZSV and three Mixograph parameters among Xiaoyan 81 and six deletion lines grown in 2013/2014^a^.

Line	Environment	ZSV (ml)	MPT (min)	MPH (%)	MPW (%)
Xiaoyan 81	ZX-13/14	22.20 ± 0.12^a^	1.62 ± 0.01^a^	58.29 ± 0.03^a^	27.30 ± 0.52^a^
	XX-13/14	23.07 ± 0.13^a^	1.59 ± 0.05^a^	60.82 ± 0.08^a^	25.31 ± 0.61^a^
DLGluA1	ZX-13/14	16.93 ± 0.07^b^	1.41 ± 0.02^b^	56.37 ± 0.09^b^	21.16 ± 0.08^b^
	XX-13/14	16.93 ± 0.07^b^	1.30 ± 0.03^b^	55.34 ± 0.46^b^	21.80 ± 1.60^b^
DLGluB1	ZX-13/14	14.93 ± 0.07^c^	1.30 ± 0.01^c^	48.58 ± 0.16^c^	16.52 ± 0.35^c^
	XX-13/14	14.80 ± 0.12^c^	1.30 ± 0.03^b^	49.95 ± 0.19^c^	17.83 ± 1.48^c^
DLGluD1	ZX-13/14	4.93 ± 0.07^e^	0.88 ± 0.01^e^	46.76 ± 0.26^d^	14.01 ± 0.77^d^
	XX-13/14	3.87 ± 0.07^e^	0.75 ± 0.01^d^	42.57 ± 0.09^d^	10.91 ± 0.78^d^
DLGluA1B1	ZX-13/14	10.93 ± 0.07^d^	1.22 ± 0.02^d^	44.07 ± 0.41^e^	15.13 ± 0.09^d^
	XX-13/14	8.13 ± 0.07^d^	1.09 ± 0.02^c^	42.58 ± 0.26^d^	11.31 ± 0.46^d^
DLGluA1D1	ZX-13/14	3.60 ± 0.12^f^	0.82 ± 0.01^f^	39.65 ± 0.34^f^	10.01 ± 0.28^e^
	XX-13/14	2.07 ± 0.07^f^	0.74 ± 0.03^d^	38.70 ± 0.35^e^	9.84 ± 0.43^d^
DLGluB1D1	ZX-13/14	2.93 ± 0.07^g^	0.69 ± 0.01^g^	31.96 ± 0.32^g^	4.33 ± 0.10^f^
	XX-13/14	1.80 ± 0.01^g^	0.59 ± 0.01^e^	28.14 ± 0.55^f^	4.38 ± 0.17^e^

^a^Each value is the mean ± SE of three independent tests. Different letters after the means indicate statistically significant difference (*P* < 0.05). MPH, midline peak height; MPT, midline peak time; MPW midline peak width; XX, Xinxiang; ZSV, Zeleny sedimentation volume; ZX, Zhaoxian.

In the three environments (BJ, ZX and XX) in 2014/2015, ZSV and two Farinograph parameters, i.e., dough development time (DDT) and dough stability time (DST), were measured. Concomitantly, a key breadmaking quality parameter, loaf volume (LV), was also examined. Higher DDT and DST values are generally associated with stronger dough^56, 57^, while a larger LV indicates better breadmaking quality^58^. From Table 2, it is apparent that the ZSV and the DDT and DST values of the six deletion lines were all significantly reduced when compared with those of Xiaoyan 81 in all three environments. Again, the strongest reduction was observed in DLGluD1, DLGluA1D1 and DLGluB1D1, with the decrease shown by DLGluA1B1 being intermediate and that by DLGluA1 and DLGluB1 being relatively low (Table 2). The changes in LV exhibited by the six deletion lines were more complex. Nevertheless, the LV values of DLGluD1, DLGluA1D1 and DLGluB1D1 were significantly and consistently lower than that of Xiaoyan 81 in all three environments (Table 2). On the other hand, significant decrease in LV was observed in only two environments for DLGluB1 and DLGluA1B1 and merely one environment for DLGluA1 (Table 2).

**Table 2:** Comparison of ZSV, two Farinograph parameters (DDT and DST) and loaf volume (LV) among Xiaoyan 81 and six deletion lines cultivated in 2014/2015^a^.

Line	Environment	ZSV (ml)	DDT (min)	DST (min)	LV (ml)
Xiaoyan 81	BJ-14/15	32.91 ± 0.17^a^	2.71 ± 0.10^a^	3.81 ± 0.13^a^	546.67 ± 21.86^a^
	ZX-14/15	30.08 ± 0.19^a^	2.72 ± 0.12 a	2.63 ± 0.08^a^	491.67 ± 16.91^a^
	XX-14/15	34.57 ± 0.07^a^	3.35 ± 0.16^a^	3.78 ± 0.13^a^	538.33 ± 46.04^a^
DLGluA1	BJ-14/15	30.24 ± 0.26^b^	2.24 ± 0.03^b^	2.66 ± 0.08^b^	520.00 ± 7.64^ab^
	ZX-14/15	27.48 ± 0.12^b^	2.69 ± 0.10^a^	2.11 ± 0.05^b^	481.67 ± 4.41^a^
	XX-14/15	29.70 ± 0.52^b^	2.89 ± 0.07^ab^	2.78 ± 0.13^b^	478.33 ± 11.67^b^
DLGluB1	BJ-14/15	22.62 ± 0.07^c^	2.18 ± 0.06^bc^	2.18 ± 0.03^c^	493.33 ± 6.67^b^
	ZX-14/15	20.29 ± 0.24^c^	1.95 ± 0.03^b^	1.83 ± 0.08^c^	461.67 ± 19.22^ab^
	XX-14/15	23.36 ± 0.25^c^	2.40 ± 0.15^b^	1.94 ± 0.08^c^	471.67 ± 4.41^b^
DLGluD1	BJ-14/15	11.77 ± 0.04^e^	1.37 ± 0.03^d^	0.82 ± 0.01^e^	468.33 ± 11.67^bc^
	ZX-14/15	11.82 ± 0.01^e^	1.48 ± 0.04^d^	0.86 ± 0.08^e^	430.00 ± 14.43^bc^
	XX-14/15	13.35 ± 0.26^e^	1.59 ± 0.08^c^	0.98 ± 0.04^d^	453.33 ± 3.33^bc^
DLGluA1B1	BJ-14/15	19.65 ± 0.03^d^	1.92 ± 0.04^c^	1.53 ± 0.02^d^	475.00 ± 0.00^b^
	ZX-14/15	15.35 ± 0.26^d^	1.77 ± 0.03^c^	1.26 ± 0.06^d^	450.00 ± 7.64^ab^
	XX-14/15	16.75 ± 0.03^d^	1.50 ± 0.06^c^	1.00 ± 0.14^d^	463.33 ± 8.82^b^
DLGluA1D1	BJ-14/15	10.35 ± 0.26^f^	1.17 ± 0.02^d^	0.76 ± 0.02^f^	438.33 ± 10.14^c^
	ZX-14/15	9.94 ± 0.02^f^	1.10 ± 0.00^e^	1.28 ± 0.14^d^	421.67 ± 8.33^bc^
	XX-14/15	9.93 ± 0.02^f^	1.23 ± 0.04^cd^	0.87 ± 0.09^d^	453.33 ± 14.81^bc^
DLGluB1D1	BJ-14/15	9.93 ± 0.03^f^	0.93 ± 0.02^e^	0.66 ± 0.03^g^	436.67 ± 8.82^c^
	ZX-14/15	9.86 ± 0.03^f^	0.90 ± 0.00^f^	0.68 ± 0.03^e^	403.33 ± 8.82^c^
	XX-14/15	8.83 ± 0.02^g^	0.89 ± 0.06^d^	0.78 ± 0.09^d^	400.00 ± 0.00^c^

^a^Each value is the mean ± SE of three separate tests. Different letters after the means indicate statistically significant difference (*P* < 0.05). BJ, Beijing; DDT, dough development time; DST, dough stability time; LV, loaf volume; XX, Xinxiang; ZSV, Zeleny sedimentation volume; ZX, Zhaoxian.

Among DLGluD1, DLGluA1D1 and DLGluB1D1, DLGluB1D1 generally exhibited the largest decreases in the measured parameters, the reductions displayed by DLGluA1D1 were intermediate, and those by DLGluD1 were comparatively smaller (Tables 1 and 2). Despite that there was only one HMW-GS (i.e., 1Ax1) left and that the examined gluten and dough functionality parameters were all severely lowered in DLGluB1D1, the LV value of this mutant was still 74.3–82.0% of that of WT control in the three environments in 2014/2015 (Table 2).

### Changes of UPP and IG

**Figure 2:**
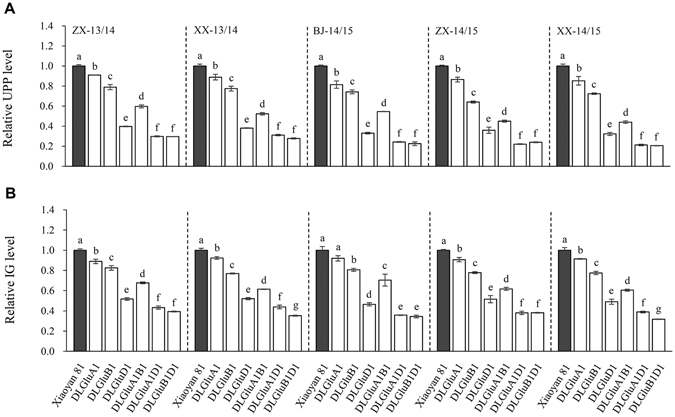
Decrease of UPP and IG in the six deletion mutants cultivated in five environments. The five environments were created by growing Xiaoyan 81 and the six deletion lines (DLGluA1, DLGluB1, DLGluD1, DLGluA1B1, DLGluA1D1 and DLGluB1D1) in two locations (ZX and XX) in 2013/2014 and three locations (BJ, ZX and XX) in 2014/2015. The values presented are means ± SE of three separate tests, with the UPP and IG levels of Xiaoyan 81 being set as 1 to facilitate comparisons. In each environment, the means marked by different letters are statistically significant (*P* < 0.05). (**A**) Relative UPP levels in Xiaoyan 81 and the six deletion mutants. (**B**) Relative IG levels in Xiaoyan 81 and the six deletion mutants. BJ, Beijing; IG, insoluble glutenin; UPP, unextractable polymeric protein; XX, Xinxiang; ZX, Zhaoxian.

The levels of UPP and IG, which indicate FGMPs^28–33^, were analyzed for Xiaoyan 81 and the six deletion lines cultivated in five environments using SE-HPLC and RP-HPLC, respectively (see Methods). Relative to Xiaoyan 81, UPP was significantly decreased in the six deletion lines in all five environments, with the magnitude of the decrease being high in DLGluD1, DLGluA1D1 and DLGluB1D1, intermediate in DLGluA1B1 and comparatively low in DLGluA1 and DLGluB1 (Fig. 2A). A similar finding was made with respect to IG for Xiaoyan 81 and the six deletion lines (Fig. 2B). Among DLGluD1, DLGluA1D1 and DLGluB1D1, DLGluB1D1 frequently exhibited the largest decreases in UPP and IG, with the reduction shown by DLGluD1 being comparatively smaller (Fig. 2). Nevertheless, there were still considerable amounts of UPP (20–30% of that of WT control, Fig. 2A) or IG (30–40% of that of WT control, Fig. 2B) present in DLGluB1D1.

A Pearson correlation analysis was conducted to investigate relationships between the levels of UPP and IG and the gluten, dough and breadmaking quality parameters presented in Tables 1 and 2. The results showed that both UPP and IG were highly significantly and positively correlated with ZSV, MPT, MPH, MPW, DDT, DST and LV, with the coefficients varying from 0.783 to 0.988 (Table 3). These data suggested that UPP and IG were equally effective in representing FGMPs, and that the different degrees of reductions in gluten, dough and breadmaking quality parameters exhibited by the six deletion lines were mainly caused by differential decreases in FGMPs.

**Table 3:** Correlation coefficients between UPP, IG and the gluten, dough and breadmaking quality parameters of the samples collected from five environments.

	ZSV	MPT	MPH	MPW	DDT	DST	LV
UPP	0.988^**^	0.948^**^	0.913^**^	0.924^**^	0.951^**^	0.947^**^	0.783^**^
IG	0.975^**^	0.949^**^	0.933^**^	0.932^**^	0.940^**^	0.924^**^	0.787^**^

DDT, dough development time; DST, dough stability time; IG, insoluble glutenin; LV, loaf volume; UPP, unextractable polymeric proteins; ZSV, Zeleny sedimentation volume. ^**^Statistically significant at *P* < 0.01.

### Investigation of HMW-GSs in IG

**Figure 3:**
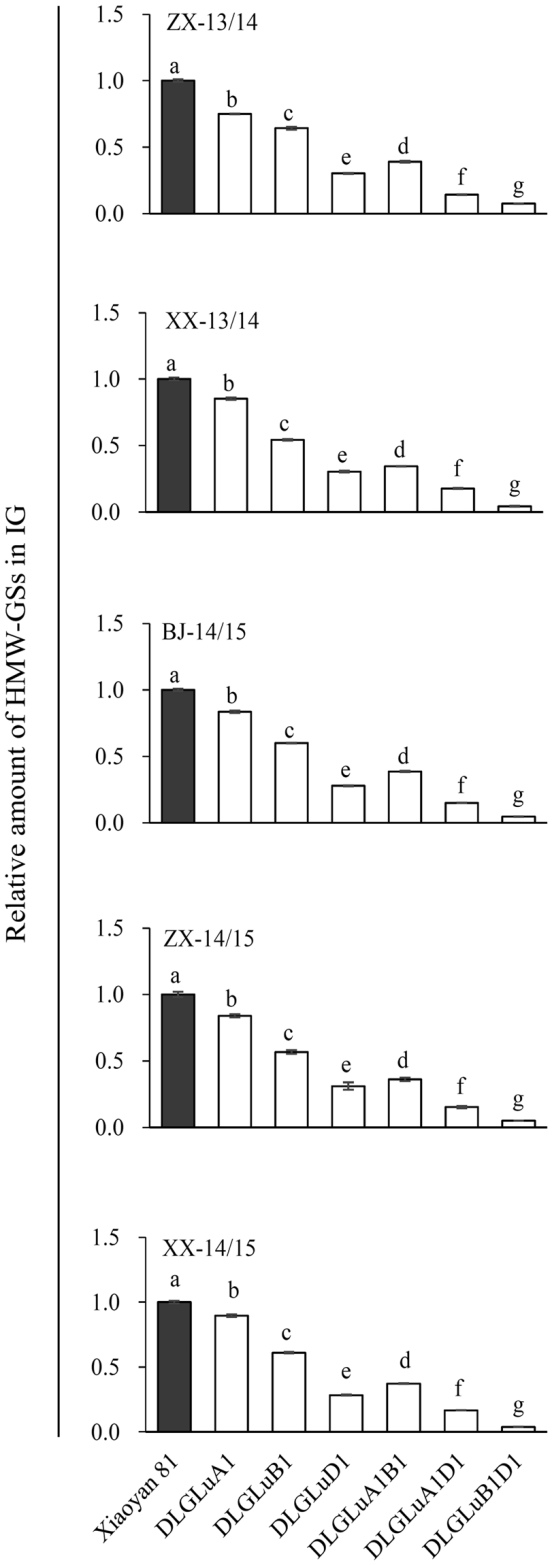
Reduction of HMW-GSs in IG in the six deletion mutants cultivated in five environments. The five environments were formed by growing Xiaoyan 81 and the six deletion lines (DLGluA1, DLGluB1, DLGluD1, DLGluA1B1, DLGluA1D1 and DLGluB1D1) in two locations (ZX and XX) in 2013/2014 and three locations (BJ, ZX and XX) in 2014/2015. The values presented are means ± SE of three separate tests, with that of Xiaoyan 81 being set as 1 to facilitate comparisons. In each environment, the means marked by different letters are statistically significant (*P* < 0.05). BJ, Beijing; HMW-GSs, high-molecular-weight glutenin subunits; IG, insoluble glutenin; XX, Xinxiang; ZX, Zhaoxian.

The level of HMW-GSs in IG was investigated for Xiaoyan 81 and the six deletion lines. As anticipated, the presence of HMW-GSs in IG was significantly reduced in all six deletion lines relative to that of Xiaoyan 81 in all five environments (Fig. 3). The reductions shown by DLGluD1, DLGluA1D1 and DLGluB1D1 were generally high, which was followed by DLGluA1B1; the decreases exhibited by DLGluA1 and DLGluB1 were comparatively low. In general, DLGluB1D1 exhibited the largest decrease, with only a minor amount of HMW-GSs (3.85–7.51% of that WT control, Fig. 3) present in IG.

The effects of lacking one or more HMW-GSs in the six deletion lines on the incorporation of the remaining HMW-GSs into IG were also examined. Generally, the lack of one or more HMW-GSs in the six deletion lines decreased the incorporation of the remaining HMW-GSs into IG (Figure S1). Such effects were most pronounced in DLGluD1, DLGluA1D1 and DLGluB1D1, with the reductions ranging from 31.9% to 74.35%. These effects were lessened in DLGluA1B1 (reductions varying from 18.8% to 32.6%), and became relatively weak in DLGluA1 and DLGluB1 (reductions ranging from 3.65% to 13.2%) (Figure S1).

### Investigation of LMW-GSs in IG and SG

**Figure 4:**
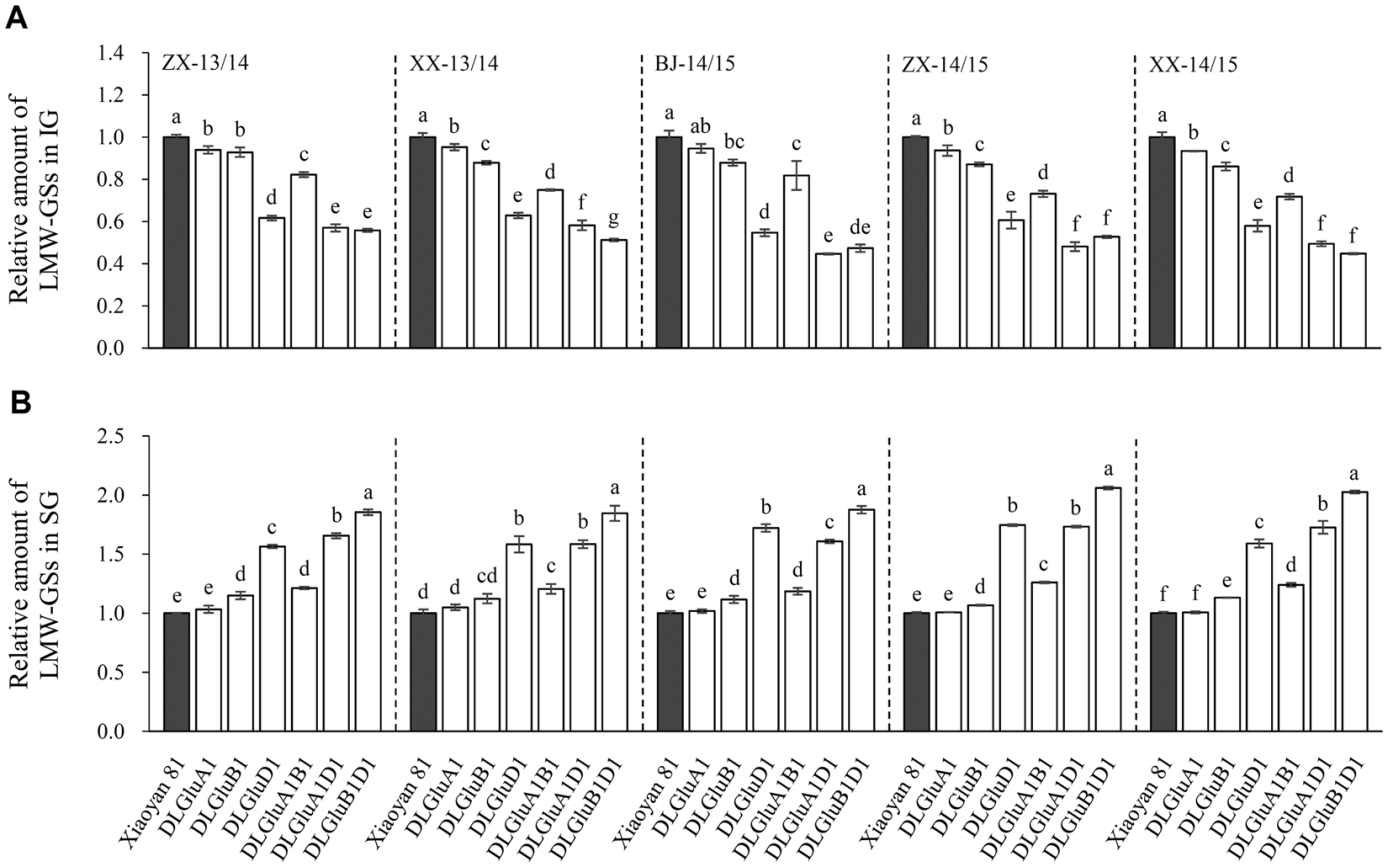
Changes in the amount of LMW-GSs in IG and SG in the six deletion mutants cultivated in five environments. Xiaoyan 81 and the six deletion lines (DLGluA1, DLGluB1, DLGluD1, DLGluA1B1, DLGluA1D1 and DLGluB1D1) were grown in two locations (ZX and XX) in 2013/2014 and three locations (BJ, ZX and XX) in 2014/2015. The resultant grain samples were analyzed for the amounts of LMW-GSs in the IG and SG fractions, respectively. The values shown are means ± SE of three separate tests, with that of Xiaoyan 81 being set as 1 to facilitate comparisons. In each environment, the means marked by different letters are statistically significant (*P* < 0.05). The amounts of LMW-GSs in IG were generally decreased in the six deletion lines compared to that in Xiaoyan 81 (**A**). On the contrary, the amounts of LMW-GSs in SG were generally increased in the six deletion lines relative to that in Xiaoyan 81 (**B**). BJ, Beijing; IG, insoluble glutenin; LMW-GSs, low-molecular-weight glutenin subunits; SG, soluble glutenin; XX, Xinxiang; ZX, Zhaoxian.

Relative to WT control, the presence of LMW-GSs in IG was consistently and most severely decreased in DLGluD1, DLGluA1D1 and DLGluB1D1 in all five environments, but this decrease was less severe in DLGluA1B1, and relatively low in DLGluA1 and DLGluB1 (Fig. 4A). Remarkably, substantial LMW-GSs (47.3–55.7% of that of WT control, Fig. 4A) were detected in the IG of DLGluB1D1, despite that there was only one HMW-GS (1Ax1) expressed in this mutant (Fig. 1).

On the contrary, the existence of LMW-GSs in SG was generally and strongly enhanced in DLGluD1, DLGluA1D1 and DLGluB1D1 in all five environments, with presence of LMW-GSs in SG increased by 57–106% in the three mutants relative to WT control (Fig. 4B). This enhancement was, however, less pronounced for DLGluA1, DLGluB1 and DLGluA1B1 (increased by 1–26% relative to WT control, Fig. 4B).

### Relative abundance of different HMW-GSs in IG

As shown in Table 4, the percentages of IG occupied by the HMW-GSs encoded by three *Glu-1* loci differed significantly, 14.79–16.38% by the *Glu-D1* subunits 1Dx2 + 1Dy12, 10.86–12.96% by the *Glu-B1* subunits 1Bx14 + 1By15, and 4.26–7.02% by the *Glu-A1* subunit 1Ax1. Obviously, the abundance of 1Dx2 + 1Dy12 in IG was substantially higher than that of 1Bx14 + 1By15 or 1Ax1, with the amount of 1Ax1 being the lowest. In line with this difference, the lack of 1Dx2 and 1Dy12 together caused the largest reduction in IG (by 47.93–53.52%), the strongest decrease of HMW-GSs in IG (69.51–72.08%), and the most severe reduction of LMW-GSs in IG (37.19–45.37%) (Table 4). The three effects were lessened when 1Bx14 and 1By15 were missed, and tended to be small when 1Ax1 was absent (Table 4).

**Table 4:** Relative abundance in IG of the HMW-GSs encoded by *Glu-A1*, *-B1* or *-D1* and their mutational effects in five environments^a^.

Environment	Subunit (*Glu-1* locus)	Abundance in IG (%)	Mutational effects		
			Decrease of IG (%)	Decrease of HMW-GSs in IG (%)	Decrease of LMW-GSs in IG (%)
ZX-13/14	1Ax1 (*-A1*)	7.02 ± 0.09^c^	11.04 ± 2.16^c^	24.89 ± 0.39^c^	6.05 ± 1.77^c^
	1Bx14 + 1By15 (*-B1*)	10.86 ± 0.21^b^	17.50 ± 2.29^b^	35.69 ± 1.22^b^	7.19 ± 2.31^b^
	1Dx2 + 1Dy12 (*-D1*)	16.22 ± 0.06^a^	48.29 ± 1.32^a^	69.67 ± 0.50^a^	38.39 ± 1.09^a^
XX-13/14	1Ax1 (*-A1*)	4.61 ± 0.37^c^	7.71 ± 1.34^c^	14.65 ± 0.15^c^	4.79 ± 1.49^c^
	1Bx14 + 1By15 (*-B1*)	12.96 ± 0.47^b^	23.04 ± 0.44^b^	45.60 ± 0.90^b^	12.19 ± 0.87^b^
	1Dx2 + 1Dy12 (*-D1*)	16.38 ± 0.45^a^	47.93 ± 1.01^a^	69.51 ± 0.99^a^	37.19 ± 1.30^a^
BJ-14/15	1Ax1 (*-A1*)	4.26 ± 0.09^c^	7.99 ± 2.61^c^	16.40 ± 0.35^c^	5.43 ± 2.13^c^
	1Bx14 + 1By15 (*-B1*)	11.16 ± 0.26^b^	19.34 ± 1.40^b^	39.79 ± 0.52^b^	12.12 ± 1.47^b^
	1Dx2 + 1Dy12 (*-D1*)	14.96 ± 0.27^a^	53.52 ± 1.52^a^	72.08 ± 0.31^a^	45.37 ± 1.58^a^
ZX-14/15	1Ax1 (*-A1*)	4.67 ± 0.05^c^	9.35 ± 2.09^c^	15.91 ± 2.87^c^	6.39 ± 2.49^c^
	1Bx14 + 1By15 (*-B1*)	11.28 ± 0.06^b^	22.22 ± 0.98^b^	43.21 ± 0.66^b^	12.92 ± 0.85^b^
	1Dx2 + 1Dy12 (*-D1*)	14.79 ± 0.42^a^	48.42 ± 3.63^a^	68.77 ± 3.35^a^	39.33 ± 3.99^a^
XX-14/15	1Ax1 (*-A1*)	4.64 ± 0.19^c^	8.60 ± 0.40^c^	10.43 ± 1.73^c^	6.63 ± 0.18^c^
	1Bx14 + 1By15 (*-B1*)	11.88 ± 0.16^b^	22.54 ± 1.59^b^	38.96 ± 0.36^b^	13.96 ± 1.89^b^
	1Dx2 + 1Dy12 (*-D1*)	15.15 ± 0.11^a^	50.97 ± 2.54^a^	71.68 ± 0.69^a^	42.04 ± 2.78^a^

^a^Each value is the mean ± SE of three separate tests. Different letters after the means indicate statistically significant difference (*P* < 0.05). HMW-GSs, high-molecular-weight glutenin subunits; IG, insoluble glutenin; LMW-GSs, low-molecular-weight glutenin subunits.

### Effects of 1Dx2 or 1Dy12 alone on gluten, dough and end-use quality parameters

The foregoing experiments highlighted the functional dominance of *Glu-D1* over *Glu-A1* and *-B1*. Because *Glu-D1* encodes both 1Dx2 and 1Dy12, it became necessary and important to examine if the two subunits may act similarly or differently in wheat end-use quality control. To this end, we compared two EMS knockout mutants (*md2-1* and *md12-1*) lacking the expression of 1Dx2 and 1Dy12, respectively (Figure S2). The two mutants were developed using the common wheat cultivar Xiaoyan 54^45^, and their genetic backgrounds were made near-identical to that of Xiaoyan 54 through six rounds of backcrossing (see Methods). Xiaoyan 54 is one of the two parents of Xiaoyan 81, and expresses an identical set of HMW-GSs as Xiaoyan 81 (Figure S2).

Xiaoyan 54 and the two knockout mutants were cultivated in two crop seasons (environments) (2014/2015 and 2015/2016), with the grains harvested being used for measuring gluten, dough and end-use quality parameters. In the two environments, ZSV and the DDT and DST values of the two knockout mutants were generally and significantly decreased relative to those of Xiaoyan 54, and in four of the six cases, the reduction exhibited by *md2-1* was significantly more severe than that by *md12-1* (Table 5). In agreement with these results, the loaf volume values of the two knockout mutants were significantly lower than those of Xiaoyan 54 in both environments (Table 5). Moreover, the loaf volume of *md2-1* tended to be smaller than that of *md12-1* (Figure S3), with the difference reached to a significant level (*P* < 0.05) in 2014/2015 (Table 5).

**Table 5:** Comparison of gluten, dough and breadmaking parameters among Xiaoyan 54, *md2-1* and *md12-1* cultivated in two crop cycles (2014/2015 and 2015/2016)^a^.

Line	Environment	ZSV (ml)	DDT (min)	DST (min)	LV (ml)
Xiaoyan 54	BJ-14/15	51.17 ± 1.66^a^	6.07 ± 0.41^a^	6.13 ± 0.85^a^	793.3 ± 4.4^a^
	BJ-15/16	48.00 ± 0.00^a^	3.06 ± 0.05^a^	3.98 ± 0.47^a^	690.0 ± 10.4^a^
*md2-1*	BJ-14/15	35.87 ± 1.80^b^	2.50 ± 0.15^c^	1.70 ± 0.20^b^	573.3 ± 11.7^c^
	BJ-15/16	30.00 ± 0.00^c^	2.49 ± 0.08^b^	1.94 ± 0.04^c^	545.0 ± 7.6^b^
*md12-1*	BJ-14/15	39.90 ± 0.36^b^	3.67 ± 0.17^b^	2.63 ± 0.34^b^	663.3 ± 19.2^b^
	BJ-15/16	36.00 ± 0.00^b^	3.00 ± 0.06^a^	2.46 ± 0.02^b^	586.7 ± 33.8^b^

^a^Each value is the mean ± SE of three separate tests. Different letters after the means indicate statistically significant difference (*P* < 0.05). BJ, Beijing; DDT, dough development time; DST, dough stability time; LV, loaf volume; ZSV, Zeleny sedimentation volume.

### Alterations in IG content and composition caused by knocking out 1Dx2 or 1Dy12

**Figure 5:**
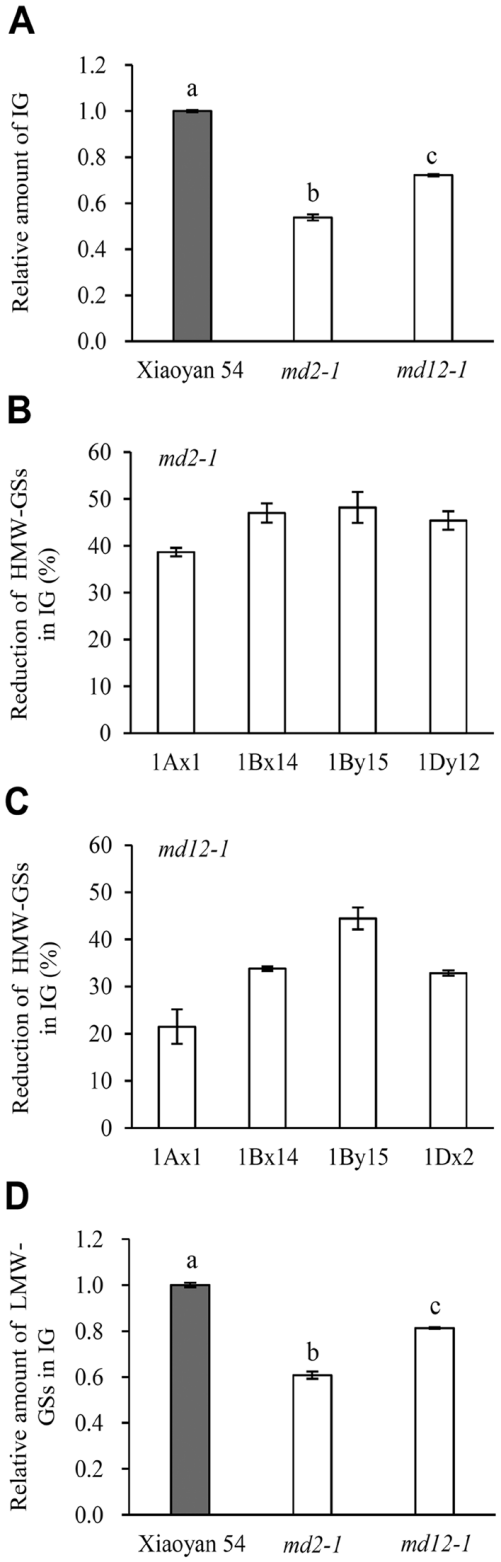
Comparative analysis of Xiaoyan 54 and two derivative knockout mutants (*md2-1* and *md12-1*) lacking the high-molecular-weight glutenin subunits (HMW-GSs) 1Dx2 and 1Dy12, respectively. The three lines were cultivated in Beijing in 2014/2015, and the resultant grain samples were collected for this set of analysis. (**A**) The amount of insoluble glutenin (IG) was decreased in *md2-1* and *md12-1* relative to that of Xiaoyan 54 (set as 1), with the decrease exhibited by *md2-1* being substantially stronger. The values shown are means ± SE of three separate tests, and those labeled by different letters are statistically significant (*P* < 0.05). (**B**) Reduction of the remaining four HMW-GSs (1Ax1, 1Bx14, 1By15 and 1Dy12) in the IG of *md2-1*. The percentages of reduction were calculated by setting the amounts of the four subunits in the IG of Xiaoyan 54 as 100%. The values shown are means ± SE of three different tests. (**C**) Reduction of the remaining four HMW-GSs (1Ax1, 1Bx14, 1By15 and 1Dx2) in the IG of *md12-1*. The percentages of reductions presented were calculated as described in (**C**). (**D**) The amount of low-molecular-weight glutenin subunits (LMW-GSs) was reduced in *md2-1* and *md12-1* relative to that of Xiaoyan 54 (set as 1), with the reduction shown by *md2-1* being considerably stronger. The values shown are means ± SE of three different tests, and those labeled by different letters are statistically significant (*P* < 0.05). The same set of analysis was also conducted using the grain samples of the three lines harvested in 2015/2016 with similar results obtained (Figure S4).

The potential consequences of lacking 1Dx2 or 1Dy12 on IG content and the incorporations of HMW-GSs and LMW-GSs into IG were investigated as described above. The results obtained for the grain samples harvested in 2014/2015 are displayed in Fig. 5. Compared with Xiaoyan 54, IG content was significantly decreased in both *md2-1* and *md12-1*, but the scale of the decrease was much higher in *md2-1* (Fig. 5A). The knockout of 1Dx2 reduced the incorporation of the remaining HMW-GSs into IG, with the percentage of the reduction being 38.7%, 47.0%, 48.2% and 45.4% for 1Ax1, 1Bx14, 1By15 and 1Dy12, respectively (Fig. 5B). The knockout of 1Dy12 also decreased the incorporation of other HMW-GSs into IG, but the percentages of the reduction observed (21.5% for 1Ax1, 33.8% for 1Bx14, 44.4% for 1By15 and 32.8% for 1Dx2) were generally lower than those caused by the lacking of 1Dx2 (compare Fig. 5B and C). Lastly, the lack of 1Dx2 decreased the incorporation of LMW-GSs in IG by 39.3%, whereas the absence of 1Dy12 reduced the incorporation of LMW-GSs in IG by only 18.7% (Fig. 5D). The results gathered for the grain samples harvested in 2015/2016 (Figure S4) were similar to those shown in Fig. 5, although the scales of the decreases in IG, LMW-GSs in IG, and the percentages of reduction of different HMW-GSs in IG tended to be smaller. These variations may be caused by differences in the growth environment between the two seasons.

## Discussion

In this work, we investigated the function of glutenin proteins in wheat end-use quality control and the mechanism involved through analyzing two series of well-defined genetic mutants. Complementary sets of data were obtained using the grain samples harvested from multiple environments, which permitted an objective assessment of the genetic effects of lacking one or two *Glu-1* loci on the examined gluten, dough and breadmaking quality parameters. The new insight obtained is discussed below.

### Comparative analysis of single and double mutants of *Glu-1* loci reinforces the dominance of *Glu-D1* in wheat end-use quality control

Previously, we found that the contribution of three *Glu-1* loci to wheat gluten and GMP parameters can be ranked as *Glu-D1* > *Glu-B1* > *Glu-A1* through analyzing single deletion mutants lacking individual *Glu-1* loci^49^. Here, we substantially extended our investigation by including both single and double deletion mutants of *Glu-1* loci, using the grain samples from multiple field environments, and testing more gluten, dough and end-use quality parameters. We consistently observed that the mutants lacking *Glu-D1* (DLGluD1) or its combination with *Glu-A1* (DLGluA1D1) or *Glu-B1* (DLGluB1D1) showed the strongest reductions in the examined gluten, dough and breadmaking quality parameters (Tables 1 and 2). These observations reinforce the dominance of *Glu-D1* in wheat end-use quality control. In most cases, the reductions displayed by DLGluA1D1 were larger than those by DLGluD1 but smaller than those by DLGluB1D1. This is consistent with the fact that the number of HMW-GSs lacked in DLGluB1D1 (i.e., 4) was more than that in DLGluA1D1 (3) or DLGluD1 (2) (Fig. 1). Clearly, there exist positive and additive interactions among the three *Glu-1* loci studied in this work, with the functional effects of the interactions between *Glu-A1* and *Glu-D1* being comparatively weaker than those between *Glu-B1* and *Glu-D1*. Past studies have also detected positive and additive interactions among the three *Glu-1* loci^47, 48, 59, 60^.

While comparing the doughs of Xiaoyan 81 and the single and double mutants using Farinograph test (Table 1), we focused on only two major parameters (DDT and DST) owing to the large number of samples needing to be assayed. However, other parameters of this test, i.e., width of Farinograph curve at peak consistency and rapidity of decline of Farinograph curve after peak consistency, also provide useful information on dough elasticity and cohesiveness^61, 62^. Upon closer examination of the Farinograph curves (Figure S5), the width of the curve in DLGluA1, DLGluB1 and DLGluA1B1 was not reduced as severely as that in DLGluD1, DLGluA1D1 and DLGluB1D1, and in general, the Farinograph curves of DLGluA1, DLGluB1 and DLGluA1B1 were declined less rapidly than those of DLGluD1, DLGluA1D1 and DLGluB1D1. Since DLGluA1, DLGluB1 and DLGluA1B1 all possessed a functional *Glu-D1*, these observations suggest that *Glu-D1* is more important than *Glu-A1* and *Glu-B1* in maintaining the width of Farinograph curve at peak consistency and for slowing down the decline of Farinograph curve after peak consistency. Because of the presence of *Glu-D1* in DLGluA1, DLGluB1 and DLGluA1B1, the functionality of the doughs of the three lines was not lowered as drastically as that of DLGluD1, DLGluA1D1 and DLGluB1D1 (all lacking *Glu-D1*). This may help to explain the less consistent decreases in LV observed for DLGluA1, DLGluB1 and DLGluA1B1 in different environments despite extensive reductions in their ZSV, DDT and DST values (Table 2).

The functional dominance of *Glu-D1* over *Glu-A1* and *Glu-B1* had also been suggested by prior studies using recombinant wheat lines differing in the composition of *Glu-1* loci and in genetic background^46–48, 63^. For example, Lawrence and coauthors demonstrated that *Glu-D1d* (a different *Glu-D1* allele encoding 1Dx5 and 1Dy10 subunits) was functionally superior to *Glu-A1* and *Glu-B1* ^46^. In contrast, our data were obtained by using six mutant lines with highly similar genetic background. Therefore, our work validated previous observation by more robust genetic data. The *Glu-D1* allele studied by us is *Glu-D1a*, which is predominant in worldwide common wheat varieties^64, 65^. Apart from *Glu-D1a* and *Glu-D1d*, there are several minor *Glu-D1* alleles (*Glu-D1b*, *-D1c*, *-D1e* and *-D1f*)^64^. It will be interesting to investigate if these minor *Glu-D1* alleles may also be functionally dominant over *Glu-A1* and *Glu-B1* in the future.

### *Glu-D1* has the strongest potency to promote the incorporation of HMW-GSs and LMW-GSs into FGMPs

FGMPs play pivotal roles in gluten and dough functionality and end-use quality^20, 66^. Their amount and polymerization characteristics are strongly affected by both the quantity and structural features of different HMW-GSs and LMW-GSs. Based on the changes in UPP, IG and the amount of HMW-GSs and LMW-GSs in IG among WT control and the six deletion mutants observed in this work (Figs 2–4), we suggest that the three *Glu-1* loci differ significantly in the ability to control the accumulation of FGMPs through promoting the incorporation of HMW-GSs and LMW-GSs into FGMPs. Specifically, *Glu-D1* has the strongest potency to promote the incorporation of HMW-GSs and LMW-GSs into FGMPs, and thus makes the largest contribution to FGMP accumulation. In contrast, *Glu-B1* is less effective than *Glu-D1*, and *Glu-A1* is weaker than *Glu-B1* in these processes. From this suggestion and the existence of highly significant correlations between the changes in gluten, dough and breadmaking quality parameters and those in UPP and IG content (Table 3), we further propose that, for individual *Glu-1* loci, the higher the potency to promote the incorporation of HMW-GSs and LMW-GSs into FGMPs, the stronger the contributions to FGMPs, gluten and dough functionality, and end-use quality performance.

The high potency of *Glu-D1* in promoting the incorporation of HMW-GSs and LMW-GSs into FGMPs is also supported by two additional lines of evidence. First, the absence of *Glu-D1* or its combination with *Glu-A1* or *Glu-B1* reduced the incorporation of the remaining HMW-GSs into IG (by 31.9–74.35%) much more strongly than that (18.8–32.6%) caused by lacking *Glu-A1*, *Glu-B1* or both (Figure S1). Second, in the absence of *Glu-D1* or its combination with *Glu-A1* or *Glu-B1*, the presence of LMW-GSs in SG was greatly enhanced (by 57–106%) relative to that (1–26%) due to the mutation of *Glu-A1*, *Glu-B1* or both (Fig. 4B).

The reason(s) underlying the enhanced potency of *Glu-D1* to promote the incorporation of HMW-GSs and LMW-GSs into FGMPs may be complex, because the subunits encoded by *Glu-D1* (1Dx2 and 1Dy12) differ from those encoded by *Glu-B1* (1Bx14 and 1By15) and *Glu-A1* (1Ax1) in multiple aspects. Nevertheless, we noticed that the abundance in IG of 1Dx2 + 1Dy12 was significantly higher than that of 1Bx14 + 1By15 or 1Ax1, and nearly equaled to the amount of 1Bx14 + 1By15 + 1Ax1 in all five environments (Table 4). Furthermore, the reduction of IG and the decreases of HMW-GSs and LMW-GSs in IG brought about by lacking 1Dx2 + 1Dy12 were always more severe than those caused by missing 1Bx14 + 1By15 or 1Ax1 (Table 4). Therefore, the high abundance of 1Dx2 + 1Dy12 in IG (relative to that of 1Bx14 + 1By15 or 1Ax1) is likely an important factor for the functional dominance of *Glu-D1* (over that of *Glu-B1* or *Glu-A1*). Because of the existence of many amino acid substitutions among the deduced proteins of 1Dx2, 1Ax1 and 1Bx14 and between those of 1Dy12 and 1By15^67, 68^, the structural differences of these subunits may also contribute to the functional dominance of *Glu-D1*. Further work is needed to validate this possibility.

### 1Dx2 has a stronger function than 1Dy12

In common wheat, *Glu-B1*, *Glu-D1* and their different alleles usually express two different HMW-GSs (one x- and one y-type)^2, 8^. Consequently, uncovering functional difference between the two subunits is essential for more comprehensively understanding the action of HMW-GSs in controlling wheat end-use quality. Some information has been gained on the function of certain HMW-GSs (e.g., 1Dx5 and 1Dy10) in controlling wheat end-use quality through studying variety population differing in HMW-GS composition, transgenic overexpression or RNA interference^69–72^. However, there is still no report on the use of knockout mutants with a near identical genetic background in investigating functional difference between the two subunits encoded by a *Glu-1* locus. In this work, we examined functional difference between the *Glu-D1* encoded subunits 1Dx2 and 1Dy12 by comparing two knockout mutants, *md2-1* (lacking 1Dx2 expression) and *md12-1* (without 1Dy12 accumulation), with their WT progenitor Xiaoyan 54. Judging from the data presented in Table 5, the function of 1Dx2 is generally and considerably stronger than that of 1Dy12 with respect to the control of the examined gluten, dough and breadmaking quality parameters. The stronger function of 1Dx2 (relative to that of 1Dy12) is most likely caused by its higher contribution to FGMPs through promoting the incorporation of more HMW-GSs and LMW-GSs into FGMPs (Fig. 5). Thus, the ability to promote the incorporation of more HMW-GSs and LMW-GSs into FGMPs is a common reason for the functional superiority of both *Glu-D1* and the 1Dx2 subunit encoded by it.

In line with our finding, earlier studies also revealed that x-type subunits had greater effects on dough functionality parameters than y-type subunits by analyzing transgenic lines and variety population^71, 73^ or through artificial incorporation of HMW-GSs into developing dough^74^. Thus, the function of x-type HMW-GSs may be generally stronger than that of y-type HMW-GSs in the control of wheat end-use quality. This raises the question what is the mechanism behind the stronger function of x-type HMW-GSs. In the current model on the structure of GMPs, y-type HMW-GSs interact covalently with LMW-GSs, with the resultant units linked by x-type HMW-GSs^43^. Although x-y and x-x linkages have been found among HMW-GSs, it is still uncertain if covalent interactions may happen between x-type HMW-GSs and LMW-GSs^23, 39, 40, 42^. We speculate that x-type HMW-GSs may interact with LMW-GSs and form FGMPs. This speculation is based on the gluten, dough and breadmaking quality parameters obtained in this work for the double mutant DLGluB1D1. Although this mutant had only one x-type HMW-GS (i.e., 1Ax1) accumulated in the grains (Fig. 1), its bread volume was still 74.3–82.0% of that of WT control (Table 2), and its UPP and IG contents were still 20–30% and 30–40% of those of WT control, respectively (Fig. 2). Moreover, a substantial amount of LMW-GSs was present in the IG of DLGluB1D1 (47.3–55.7% of that of WT control, Fig. 4). Considering that there was no y-type HMW-GS present in DLGluB1D1, and the level of 1Ax1 in its grains was rather low (Fig. 3 and Figure S1), the interactions between 1Ax1 and LMW-GSs, if existed, may be fairly effective. Therefore, the actions of x-type HMW-GSs in FGMP formation are likely more extensive than currently thought. A better elucidation of these actions may help to explain the functional superiority of x-type HMW-GSs (over their y-type counterparts) in wheat-end use quality control.

In summary, we have generated new information on the functional difference among three *Glu-1* loci and between two HMW-GSs (1Dx2 and 1Dy12). The three loci, as well as the two subunits, differ significantly in the efficacy to promote the incorporation of HMW-GSs and LMW-GSs into FGMPs, and these differences are largely responsible for the functional dominance of *Glu-D1* over *Glu-A1* and *Glu-B1* and the functional superiority of 1Dx2 to 1Dy12. This insight increases our understanding of the function of HMW-GSs in controlling important gluten and dough properties and breadmaking performance. Moreover, the data from this work and our previous study^45^ confirm that the *Glu-1* locus deletion mutants and the EMS mutants lacking individual or combinations of HMW-GSs are valid materials for further research on wheat end-use quality. Continued analysis of these mutants with functional genomics approaches (e.g., using transcriptomic, proteomic and/or metabolic methods) may shed new light on the genetic and molecular basis of gluten and dough functionalities and lead to valuable strategies for improving wheat end-use traits.

## Methods

### Plant materials and growth conditions

Genetic crosses were conducted in between DLGluA1, DLGluB1 and DLGluD1, which have *Glu-A1*, *-B1* and *-D1* deleted, respectively^49^. Homozygous plants missing two *Glu-1* loci (*Glu-A1* and *-B1*, *Glu-A1* and *-D1* or *Glu-B1* and *-D1*) were identified by checking HMW-GS composition in F2 seeds, and used to develop the three double deletion mutants DLGluA1B1, DLGluA1D1 and DLGluB1D1. Xiaoyan 81 (WT progenitor) and the six deletion mutants were grown in five field environments with normal supplies of irrigation water and chemical fertilizers^75^. The five environments were created by growing the materials in two locations (Zhaoxian and Xinxiang) in 2013/2014 and three locations (Beijing, Zhaoxian and Xinxiang) in 2014/2015. The knockout mutants *md2-1* and *md12-1*, lacking the expression of 1Dx2 and 1Dy12, respectively, were backcrossed six times using their WT progenitor Xiaoyan 54 as recurrent parent^45^. In this study, the three isogenic lines (Xiaoyan 54, *md2-1* and *md12-1*) were cultivated in Beijing in two wheat crop cycles (2014/2015 and 2015/2016) as described above. The deletion mutants and their grains were checked for agronomic traits using standard methods^49, 75^. Flour samples were prepared for the different lines as reported before^49^.

### SDS-PAGE

HMW-GSs accumulated in the different experimental lines were extracted using 20 mg flour, and separated with 10% SDS-PAGE according to the method describe previoulsy^76^.

### RP-HPLC

IG and SG levels in the flour samples were assayed using RP-HPLC. The majority of the analysis steps were carried out at room temperature (RT, approximately 25 °C) except where noted. IG and SG were extracted following the method detailed previously^31^. Briefly, for each line (WT control or deletion mutant), the flour sample (50 mg) was extracted twice with 0.5 ml of 50% (v/v) 1-propanol. After each extraction, the sample was centrifuged at 2,200 g for 3 min. The pellet was washed with 0.5 ml 50% (v/v) 1-propanol for 1 min and centrifuged at 15,000 g for 3 min. The pellet was further extracted with 0.5 ml 50% (v/v) 1-propanol containing 1% (w/v) dithiothreitol (DTT) at 65 °C for 1 h, followed by the addition of 1.4% (v/v) 4-vinylpyridine for 30 min at 65 °C. The mixture was then centrifuged at 15,000 g for 10 min, with the supernatant retained as IG fraction. The three supernatants after the extraction with 50% (v/v) 1-propanol in the preceding steps were combined, and 1-propanol was added to 70%. After centrifugation at 12,000 g for 3 min, the precipitated proteins were dissolved in 0.5 ml 50% (v/v) 1-propanol containing 1% (w/v) DTT by incubating at 65 °C for 1 h. Subsequently, 4-vinylpyridine was added to 1.4% (v/v), and the mixture was maintained at 65 °C for another 30 min. Lastly, the mixture was centrifuged at 15,000 g for 10 min, with the supernatant kept as SG fraction. The SG and IG fractions were all filtered through 0.45 μm nylon filter before being analyzed by RP-HPLC. RP-HPLC analysis was accomplished with the Agilent 1260 infinity Quaternary LC System using a C18 column. The elution conditions were essentially those described by González-Torralba and coauthors^77^. For each RP-HPLC run, a volume of 15 μl of the filtered IG (SG) was analyzed. The amount of HMW-GSs and LMW-GSs were calculated by integrating the areas under the corresponding protein peaks of the chromatogram.

### SE-HPLC

The assay of UPP content by SE-HPLC was conducted at RT following the method described in a previous study^78^. Briefly, for each sample to be assayed for UPP, 10 mg flour was suspended in 1 ml extraction buffer (50 mM sodium phosphate containing 0.5% SDS, pH 6.9), and vortexed for 10 min. The mixture was centrifuged at 17,000 g for 15 min, and the resultant pellet was resuspended in 1 ml extraction buffer, followed by sonication in a SCIENTZ-IID sonicator (Scientz Biotechnology Co., Ningbo, China). The condition of sonication was 20% output power for 30 s using a 3 mm sonicator probe, with the probe placed at 1/3 distance from the bottom of the microfuge tube. Afterwards, the mixture was centrifuged at 17,000 g for 15 min, with the supernatant retained as UPP. It was filtered through 0.45 μm nylon filter, and analyzed by SE-HPLC with the Agilent 1260 infinity Quaternary LC System using a Biosep SEC-4000 column (Phenomenex, Torrence, CA, USA). For each filtered UPP sample, an aliquot of 15 μl was assayed by SE-HPLC, with UPP content calculated by integrating the areas under the corresponding peaks of the chromatogram.

### Evaluation of ZSV and Mixograph, Farinograph and loaf volume parameters

ZSV was measured following the method described previously^52^. Mixograph parameters (MPT, MPH and MPW) were determined with a 10 g mixograph system (National Manufacturing Co., Lincoln, NE, USA) using the AACCI method 54–40.02^79^. Farinograph parameters (DDT and DST) and loaf volume were measured according to the AACCI methods 54–21.02 and 10–10.03, respectively^79^.

### Statistical analysis

For the experiments described above, three separate tests were carried out for each sample. The data obtained were subjected to one-way analysis of variance (ANOVA) using IBM SPSS Statistics 19 software (IBM, New York, USA), followed by the least significant difference multiple comparison test. Pearson’s correlation coefficients between IG, UPP and the gluten, dough and breadmaking quality parameters were calculated using the IBM SPSS Statistics 19 software.

## Electronic supplementary material

New insight into the function of wheat glutenin proteins as investigated with two series of genetic mutants
