# Supplementary material for: New insight into the function of wheat glutenin proteins as investigated with two series of genetic mutants
Source: Sci Rep. 2017 Jun 13;7:3428. doi: 10.1038/s41598-017-03393-6 (PMC5469833; doi:10.1038/s41598-017-03393-6)
Supplement: Supplementary file 1 — New insight into the function of wheat glutenin proteins as investigated with two series of genetic mutants [file 41598_2017_3393_MOESM1_ESM.doc]

**New insight into the function of wheat glutenin proteins as investigated with two series of genetic mutants**

Zhaojun Wang^1,2^, Yiwen Li^1^, Yushuang Yang^1,2^, Xin Liu^1^, Huanju Qin^1^, Zhenying Dong^1^, Shuhai Zheng^3^,Kunpu Zhang^1*^ & Daowen Wang^1,4*^

^1^The State Key Laboratory of Plant Cell and Chromosome Engineering, Institute of Genetics and Developmental Biology, Chinese Academy of Sciences, Beijing 100101, China. ^2^University of Chinese Academy of Sciences, Beijing 100049, China. ^3^Zhaoxian Institute of Agricultural Sciences, Zhaoxian 051530, China. ^4^The Collaborative Innovation Center for Grain Crops, Henan Agricultural University, Zhengzhou 450002, China.

^*^Corresponding authors: Kunpu Zhang (kpzhang@genetics.ac.cn) & Daowen Wang (dwwang@genetics.ac.cn).

**Figure S1.** Effects of lacking one or more *Glu-1* loci and the encoded high-molecular-weight glutenin subunits (HMW-GSs) on the incorporation of the remaining HMW-GSs into insoluble glutenin (IG). The values shown are percentages of decreases of the remaining HMW-GSs in IG for the six deletion lines (DLGluA1, DLGluB1, DLGluD1, DLGluA1B1, DLGluA1D1 and DLGluB1D1). These values were calculated by setting the amounts of the corresponding subunits in the IG of wild type control (Xiaoyan 81) as 100%. Each value was the mean ± SE of three separate tests using different batches of grains. The subunits lacked in the six deletion lines are indicated in the brackets. The data depicted were obtained using the grains harvested from Xinxiang in the crop cycle 2014/2015, and are representative of those gathered for the other four environments.

**Figure S2.** Composition of high-molecular-weight glutenin subunits in Xiaoyan 81, Xiaoyan 54, *md2-1* and *md12-1*. Xiaoyan 81 and Xiaoyan 54 express an identical set of subunits (1Ax1, 1Bx14, 1By15 and 1Dx2). The two knockout mutants (*md2-1* and *md12-1*), identified from an ethyl methanesulfonate mutated population of Xiaoyan 54, lack the 1Dx2 and 1Dy12 subunits, respectively.

**Figure S3.** Comparison of the bread loaves of Xiaoyan 54, *md2-1* and *md12-1*. Relative to Xiaoyan 54, the loaf volume was substantially reduced in *md2-1* and *md12-1* that lacked the high-molecular-weight glutenin subunits 1Dx2 and 1Dy12, respectively, with the scale of the reduction being much higher in *md2-1*. The data shown were obtained using the grain samples harvested in the crop cycle 2014/2015. Similar results were produced with the grain samples harvested in the crop cycle 2015/2016.

**Figure S4.** Comparative analysis of Xiaoyan 54 and the two knockout mutants (*md2-1* and *md12-1*) lacking the high-molecular-weight glutenin subunits (HMW-GSs) 1Dx2 and 1Dy12, respectively. The three lines were grown in Beijing in the crop cycle 2015/2016, and resultant grain samples were collected for this set of analysis. (**A**) The amount of insoluble glutenin (IG) was decreased in *md2-1* and *md12-1* relative to that of Xiaoyan 54 (artificially set as 1), with the decrease exhibited by *md2-1* being substantially stronger. The values shown were means ± SE of three separate tests, and those labeled by different letters were statistically significant (*P* < 0.05). (**B**) Reduction of the remaining four HMW-GSs (1Ax1, 1Bx14, 1By15 and 1Dy12) in the IG of *md2-1*. The percentages of reduction were calculated by setting the amounts of the four subunits in the IG of Xiaoyan 54 as 100%. The values shown were means ± SE of three different tests. (**C**) Reduction of the remaining four HMW-GSs (1Ax1, 1Bx14, 1By15 and 1Dx2) in the IG of *md12-1*. The percentages of reductions presented were obtained as described above. (**D**) The amount of low-molecular-weight glutenin subunits (LMW-GSs) was reduced in *md2-1* and *md12-1* relative to that of Xiaoyan 54 (artificially set as 1), with the reduction shown by *md2-1* being considerably stronger. The values shown were means ± SE of three different tests, and those labeled by different letters were statistically significant (*P* < 0.05).

**Figure S5.** Farinograph curves for Xiaoyan 81 and six deletion lines. To facilitate data interpretation, a model Farinograph curve is provided on the top leaf corner. Sample names are shown in the upper right corner of each curve. Compared with Xiaoyan 81, the DDT and DST values were decreased for all six deletion lines. Curves for those lines lacking *Glu-D1* (DLGluD1, DLGluA1D1 and DLGluB1D1) reached the peak in very short time, and dropped much quicker than the other lines, and their band width was also much thinner. The data shown were obtained using the grains harvested from Xinxiang in the crop cycle 2014/2015, and are representative of those obtained for the other two environments.

**Table S1.** Comparison of important agronomic traits among Xiaoyan 81 and six deletion lines cultivated in three environments in the crop cycle 2014/2015.

**Figure S1**


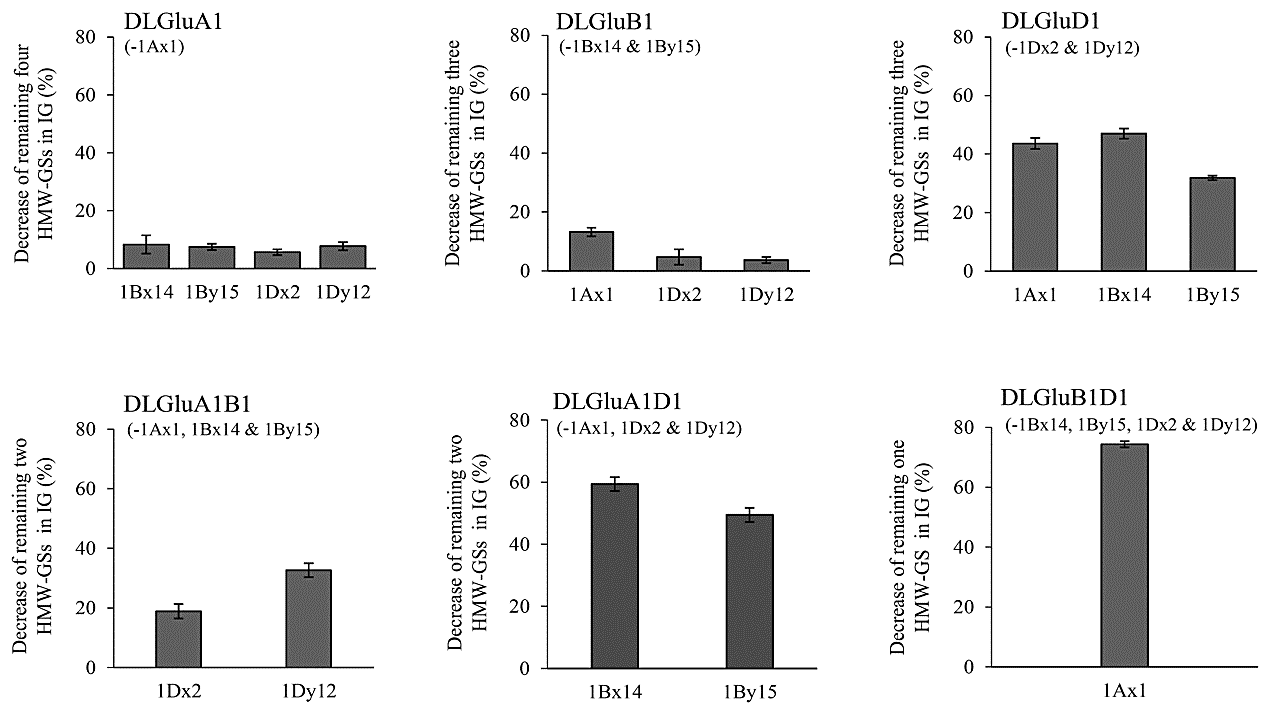


**Figure S2**


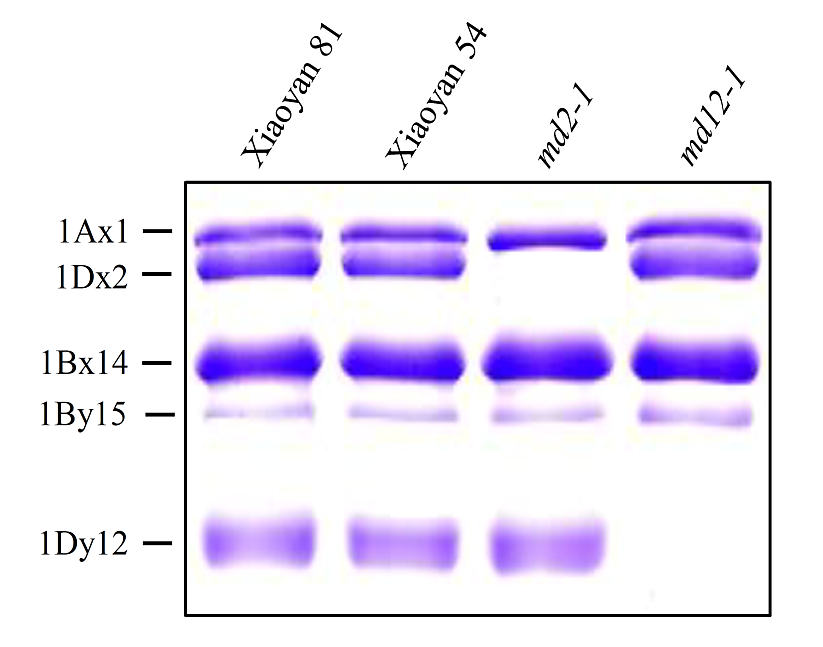


**Figure S3**

**
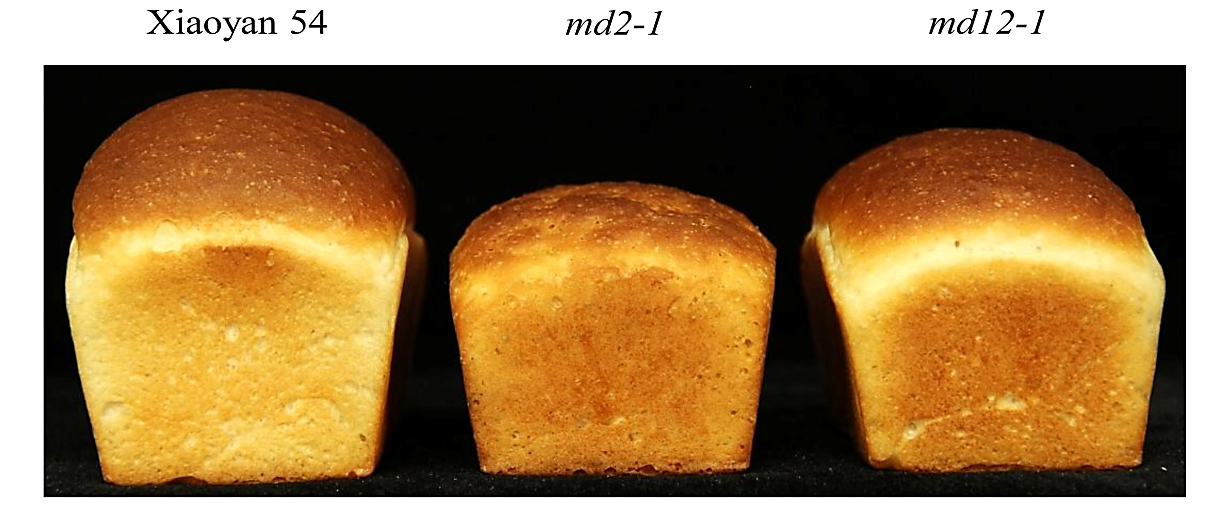
**

**Figure S4**


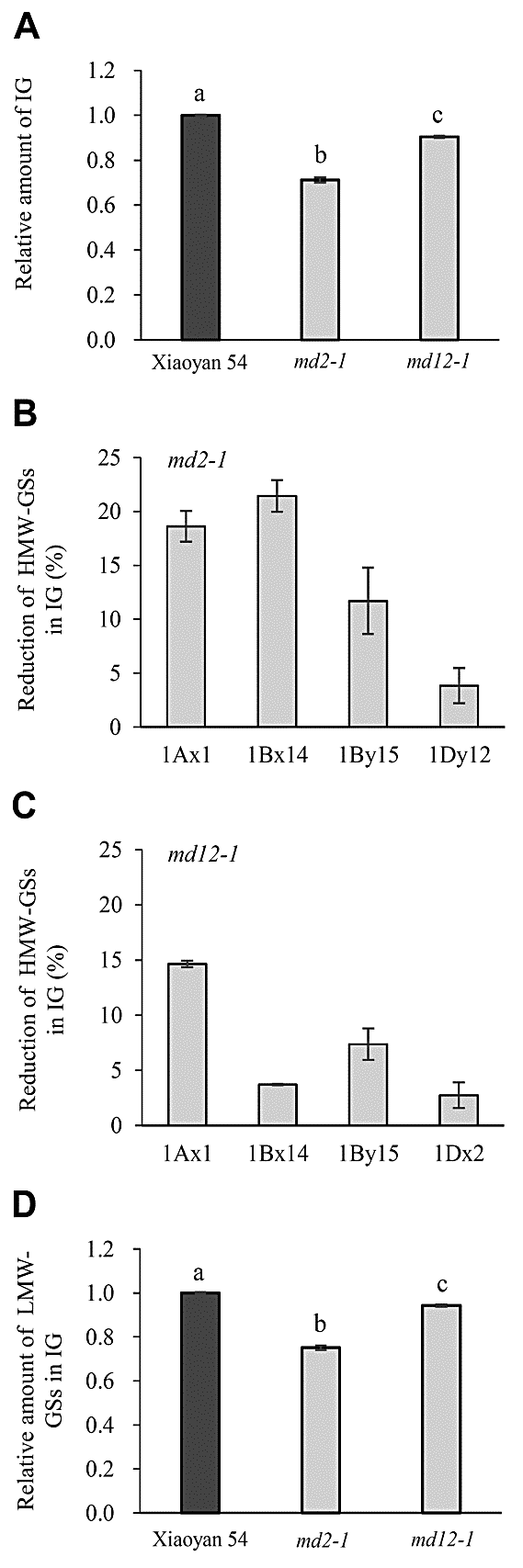


**Figure S5**


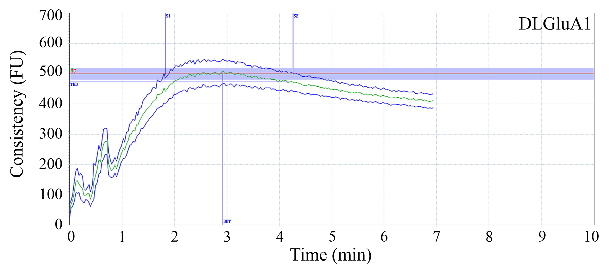

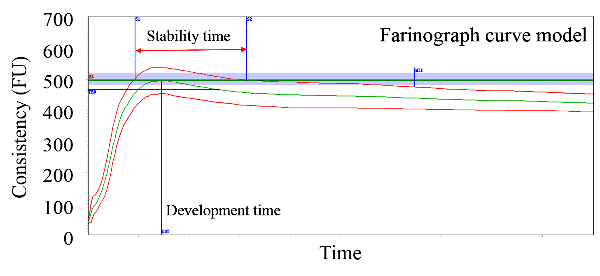

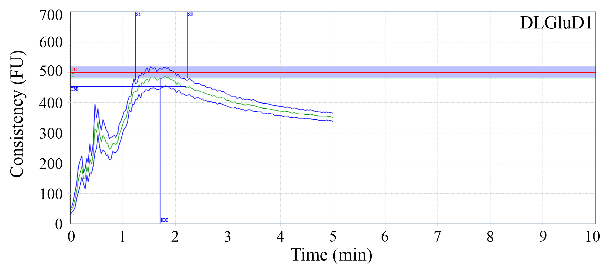

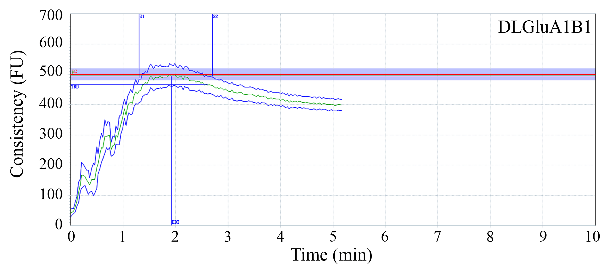

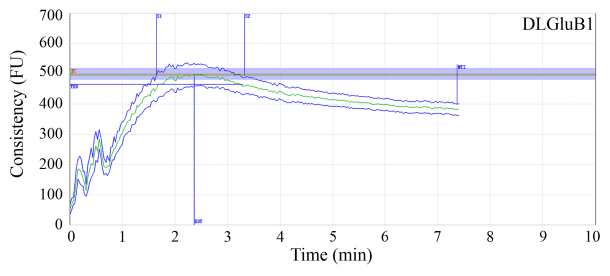

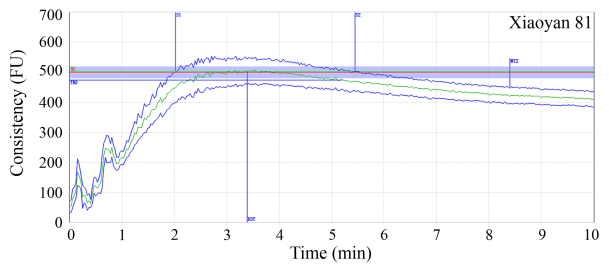

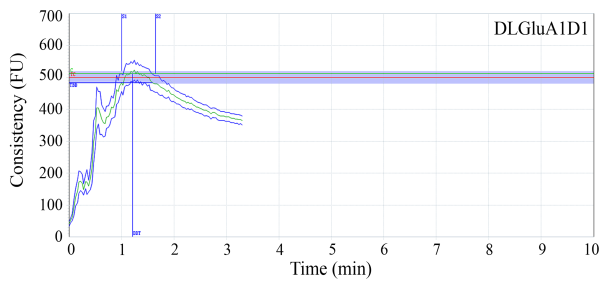

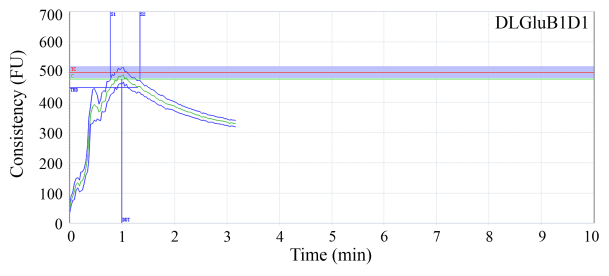


**Table S1.** Comparison of important agronomic traits among Xiaoyan 81 and six deletion lines cultivated in three environments in the crop cycle 2014/2015 ^a^.

| **Line** | **Environment** | **Plant height (cm) ^b^** | **Tiller number ^b^** | **Kernel per spike ^b^** | **Grain length (mm) ^c^** | **Grain width (mm) ^c^** | **Thousand kernel**  **weight (g) ^c^** |
| --- | --- | --- | --- | --- | --- | --- | --- |
| Xiaoyan 81 | BJ-14/15 | 57.22 ± 0.57 a | 11.56 ± 1.71 a | 50.44 ± 2.22 a | 6.41 ± 0.01 a | 2.99 ± 0.04 a | 36.06 ± 0.70 a |
|  | ZX-14/15 | 56.67 ± 1.05 a | 11.78 ± 0.76 a | 45.56 ± 2.03 a | 6.16 ± 0.04 a | 3.01 ± 0.02 a | 35.93 ± 0.51 a |
|  | XX-14/15 | 75.78 ± 1.08 a | 10.56 ± 0.63 a | 43.89 ± 2.50 a | 6.06 ± 0.03 a | 3.05 ± 0.05 a | 39.37 ± 0.47 a |
| DLGluA1 | BJ-14/15 | 56.11 ± 1.12 a | 13.56 ± 1.11 a | 46.11 ± 3.07 a | 6.29 ± 0.06 a | 2.99 ± 0.02 a | 35.20 ± 0.54 a |
|  | ZX-14/15 | 54.67 ± 1.87 a | 11.56 ± 0.99 a | 49.67 ± 2.15 a | 6.17 ± 0.06 a | 3.00 ± 0.01 a | 34.81 ± 0.53 a |
|  | XX-14/15 | 74.78 ± 1.66 a | 12.78 ± 1.95 a | 41.67 ± 2.33 a | 6.04 ± 0.03 a | 3.06 ± 0.04 a | 39.60 ± 0.57 a |
| DLGluB1 | BJ-14/15 | 57.44 ± 0.53 a | 10.89 ± 2.21 a | 46.44 ± 2.97 a | 6.39 ± 0.05 a | 2.99 ± 0.01 a | 35.78 ± 0.56 a |
|  | ZX-14/15 | 54.89 ± 1.16 a | 12.22 ± 1.27 a | 49.67 ± 2.42 a | 6.20 ± 0.00 a | 2.96 ± 0.06 a | 34.16 ± 0.85 a |
|  | XX-14/15 | 76.44 ± 1.17 a | 12.00 ± 1.20 a | 41.11 ± 1.70 a | 6.08 ± 0.01 a | 3.03 ± 0.02 a | 38.86 ± 0.30 a |
| DLGluD1 | BJ-14/15 | 56.56 ± 0.99 a | 10.78 ± 1.58 a | 49.56 ± 2.26 a | 6.31 ± 0.06 a | 2.96 ± 0.02 a | 35.60 ± 0.26 a |
|  | ZX-14/15 | 53.67 ± 1.72 a | 10.00 ± 1.12 a | 45.22 ± 1.75 a | 6.19 ± 0.02 a | 3.03 ± 0.03 a | 35.87 ± 0.44 a |
|  | XX-14/15 | 74.56 ± 0.71 a | 13.22 ± 1.40 a | 39.89 ± 2.78 a | 6.08 ± 0.02 a | 3.06 ± 0.03 a | 39.29 ± 0.39 a |
| DLGluA1B1 | BJ-14/15 | 55.22 ± 0.85 a | 11.11 ± 1.42 a | 49.56 ± 2.30 a | 6.39 ± 0.04 a | 2.96 ± 0.02 a | 35.65 ± 0.76 a |
|  | ZX-14/15 | 53.22 ± 1.16 a | 12.00 ± 1.00 a | 50.67 ± 2.29 a | 6.23 ± 0.02 a | 2.99 ± 0.05 a | 34.76 ± 1.01 a |
|  | XX-14/15 | 73.78 ± 1.01 a | 11.00 ± 1.34 a | 42.22 ± 1.16 a | 6.03 ± 0.02 a | 2.99 ± 0.03 a | 38.11 ± 0.80 a |
| DLGluA1D1 | BJ-14/15 | 57.44 ± 0.58 a | 12.78 ± 1.75 a | 50.89 ± 2.45 a | 6.40 ± 0.05 a | 2.97 ± 0.03 a | 35.67 ± 1.23 a |
|  | ZX-14/15 | 55.56 ± 1.14 a | 10.56 ± 0.87 a | 50.78 ± 2.27 a | 6.23 ± 0.05 a | 3.00 ± 0.02 a | 35.30 ± 0.74 a |
|  | XX-14/15 | 76.78 ± 0.91 a | 12.56 ± 1.48 a | 43.89 ± 1.84 a | 6.10 ±0.03 a | 3.04 ± 0.02 a | 38.39 ± 0.83 a |
| DLGluB1D1 | BJ-14/15 | 57.22 ± 1.20 a | 12.67 ± 1.55 a | 48.11 ± 1.87 a | 6.41 ± 0.05 a | 2.93 ± 0.02 a | 35.86 ± 0.63 a |
|  | ZX-14/15 | 56.78 ± 0.74 a | 10.89 ± 0.70 a | 48.33 ± 2.00 a | 6.24 ± 0.05 a | 2.98 ± 0.06 a | 35.44 ± 0.24 a |
|  | XX-14/15 | 74.33 ± 0.97 a | 10.00 ± 1.35 a | 44.44 ± 2.15 a | 6.07 ± 0.03 a | 3.05 ± 0.00 a | 38.96 ± 0.27 a |

^a^ Values are present as means ± SE. No significant difference was found among Xiaoyan 81 and six deletion lines in the six assessed traits in three environments. BJ, Beijing; XX, Xinxiang; ZX, Zhaoxian.

^b^ Plant height, tiller number and kernel per spike traits were each determined using 30 mature plants (after grain filling and immediately before harvesting).

^c^ Grain length, grain width and thousand kernel weight traits were each measured with the seeds harvested from three separate plots (three rows, 2 m per row for each plot).
